# Characterization, in-silico, and in-vitro study of a new steroid derivative from *Ophiocoma dentata* as a potential treatment for COVID-19

**DOI:** 10.1038/s41598-022-09809-2

**Published:** 2022-04-07

**Authors:** Mohamed S. M. Abd El Hafez, Miral G. AbdEl-Wahab, Mohamed G. Seadawy, Mostafa F. El-Hosseny, Osama Beskales, Ali Saber Ali Abdel-Hamid, Maha A. El Demellawy, Doaa A. Ghareeb

**Affiliations:** 1 National Institute of Oceanography and Fisheries (NIOF), Cairo, Egypt; 2 Center of Excellence for Drug Preclinical Studies (CE-DPS), Pharmaceutical and Fermentation Industries Development Centre (PFIDC), City of Scientific Research and Technological Applications (SRTA-City), New Borg El Arab, Egypt; 3 Biological Prevention Department, Egyptian Army, Cairo, Egypt; 4 Medical Services Department, The Egyptian Army, Cairo, Egypt; 5 Medical Biotechnology Department, Genetic Engineering & Biotechnology Research Institute, City of Scientific Research and Technological Applications (SRTA-City), New Borg El Arab, Egypt; 6 Bio-screening and Preclinical Trial Lab, Biochemistry Department, Faculty of Science, Alexandria University, Alexandria, Egypt; 7 Biochemistry Department, Faculty of Science, Alexandria University, Alexandria, Egypt

**Keywords:** Biochemistry, Biotechnology

## Abstract

The medicinal potential of marine invertebrates' bioactive components that may act as anti-COVID-19 demonstrated promising results. *Ophiocoma dentata*, which is common in the Red Sea, is one such source. Therefore, this study aimed to isolate a new compound from the brittle star, *Ophiocoma dentata*, and evaluate its efficacy as anti-COVID-19 in-silico and in-vitro. Standard procedures were followed in order to assess the isolated compound’s preliminary toxicity and anti-inflammatory properties. Computer virtual screening technology through molecular docking and ADMET studies was conducted as well as a new steroid derivative was isolated for the first time, named 5α-cholesta-4(27), 24-dien-3β, 23 β-diol. Investigation of the Anti-Covid-19 activity of the isolated compound using a Plaque reduction assay revealed 95% inhibition at a concentration of 5 ng/µl (12.48 µM). Moreover, this compound showed an IC_50_ of 11,350 ± 1500 ng/ml against the normal fibroblast cells, indicating its safety. Interestingly, this compound exhibited anti-inflammatory activity with an IC_50_ of 51.92 ± 0.03 μg/ml compared to a reference drug’s IC_50_ of 53.64 ± 0.01 μg/ml, indicating that this compound is a potent anti-inflammatory. In silico data have proved that the isolated compound is a promising viral inhibitor against SARS-CoV2 and is thus recommended as a future nature preventive and curative antiviral drug.

## Introduction

More than 5 million people have died due to the COVID-19 outbreak worldwide, which has inflicted political and socioeconomic disruptions in our daily lives^1^. Researchers have started repurposing already FDA-approved medications for COVID-19 treatment as a quick method to reduce the time required for safety and approval studies^2^. Currently, scientific efforts focus on developing a suitable medication from the old traditional pharmaceuticals used for earlier coronavirus strains^3^. Many natural product derivative treatments have been reported to prevent the entry and replication of various coronaviruses, such as SARS and MERS. Natural compounds and their molecular frameworks have long been considered valuable starting points or sources for drug discovery^4^.

Blunt et al.^5^ revealed that marine natural products are beneficial substances for drug discovery due to their wide variety of bioactivities, including anti-infective, anti-proliferative, antibiotic, and anti-tumor properties. Marine invertebrates generally are motionless and stuck to the ocean bottom, they use potent secondary metabolites to prevent the growth of invading neighbors and attract food. Such survival conditions stimulated the production of a highly abundant variety of biologically active substances^5^. Echinoderms demonstrate as an untapped source in an attempt to identify novel and beneficial products^6^. According to Fayed et al.^7^, marine natural products have demonstrated antiviral activity against coronaviruses, and various aquatic species have previously been discovered to produce a variety of antiviral lead compounds. Brittle stars (Ophiuroidea) are the largest echinoderm group in terms of species, as well as the most frequent^8^. There have been a few studies on the biological activity of brittle star species have been published. However, the antiviral activity of the brittle star revealed that the most abundant bioactive metabolites derived from brittle star are steroidal glycosides, carotenoid sulfates, and naphthoquinone. All of which are essential for life maintenance on earth and have the potential to be used in biomedicine^8^.

Molecular modeling and virtual screening are examples of computational-based techniques that contribute to understanding the molecular components of protein–ligand interactions throughout the rational drug discovery process^9,10^. Two intriguing therapeutic targets for SARS-CoV-2 related disorders are the main protease (Mpro) and the RNA-dependent RNA polymerase (RdRP), which are responsible for the viral polyprotein proteolytic process along with viral genome replication and transcription^11,12^. These two target sites have been intensively docked to develop or distinguish structure-based effective medicines for COVID-19 based on their critical role in the life cycle of SARS-CoV-2^13^. Natural bioactive compounds are currently being screened for their affinity for COVID-19 molecular targets using molecular docking, with the advantage that natural products are free of harmful or adverse effects^14,15^. This study aims to isolate a new compound from the brittle stars, *Ophiocoma dentata* that can resist COVID-19*. *In addition, structure elucidation, anti-inflammatory activity, molecular docking and in silico ADMET studies were performed.

## Results

### Structure determination of the isolated compound

The new compound was obtained as yellow crystals, and HREIMS provided its molecular formula, C_27_H_44_O_2_ (m/z 400.6470), indicating the presence of six degrees of unsaturation. In addition to the double bond (1742 cm^−1^), the FTIR spectrum revealed the presence of hydroxyl (3424 cm^−1^). ^1^H NMR spectrum of the isolated compound showed the presence of one proton multiplet at δ 4.14, the position and multiplicity of which was indicative of H-3 of the steroidal nucleus. Presence of four methyl [δ 0.86 (3H, s, H-18), δ 1.19 (3H, s, H-19), δ 1.06 (3H, d, J = 10 Hz, H-21), δ 5.93 (3H, m, H-24)] and four-terminal olefinic protons [δ 5.09 and δ 4.93 (H-25 and H-27)].

**Figure 1:**
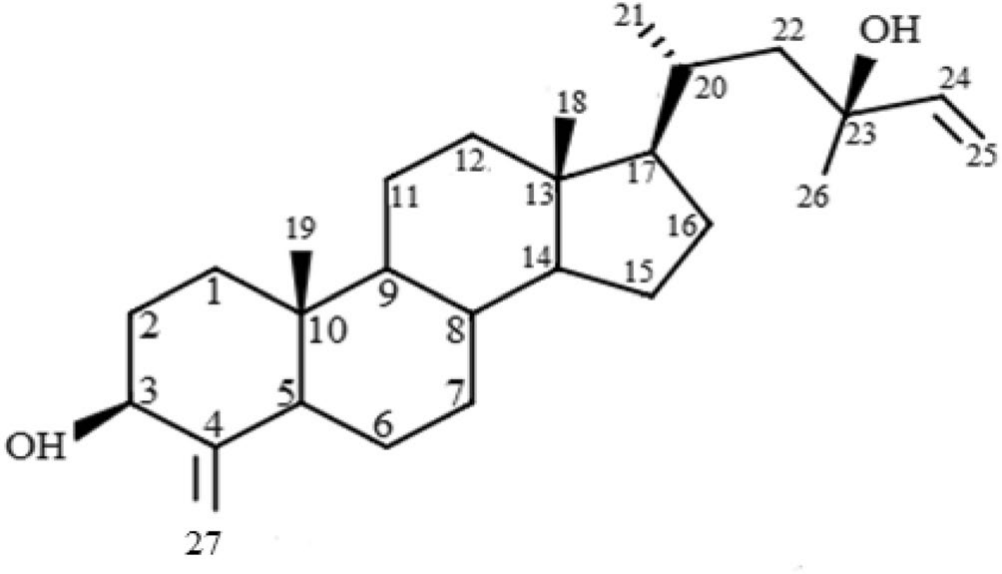
5α-cholesta-4(27), 24-dien-3β, 23 β-diol isolated from the brittle star (*O. dentata*).

The ^13^C NMR spectrum of the isolated compound revealed the resonance of 27 carbons identified as four methyls, ten methylene (including two terminal double bonds), one oxygenated methine, and five quaternary carbons. The isolated compound's complete ^1^H and ^13^C NMR chemical shifts were assigned using a combination of COSY-45°, HSQC, and HMBC spectra. These spectroscopic studies suggested that the isolated compound had a steroidal type skeleton. Consequently, were proposed the structure for this new compound named 5α-cholesta-4(27), 24-dien-3β, 23 β-diol. ^1^H NMR, ^13^C NMR, HSQC, HMBC, COSY, NOESY, and FTIR data are depicted in (Fig. 1 and supplementary Figs. S2–S8; Table S1).

### Molecular docking, ADMET analysis, and in silico toxicity studies of the isolated compound

In this work, the binding potential of the isolated compound against three proteins of SARS-CoV-2 has been investigated. The selected proteins are: (i) COVID-19 main protease (M^pro^) (PDB ID: *6*lu7, resolution: 2.16 Å), (ii) nonstructural protein (nsp)10 (PDB ID: 6W4H, resolution: 1.80 Å), and (iii) RNA-dependent RNA polymerase (PDB ID: 7BV2, resolution: 2.50 Å).

**Figure 2:**
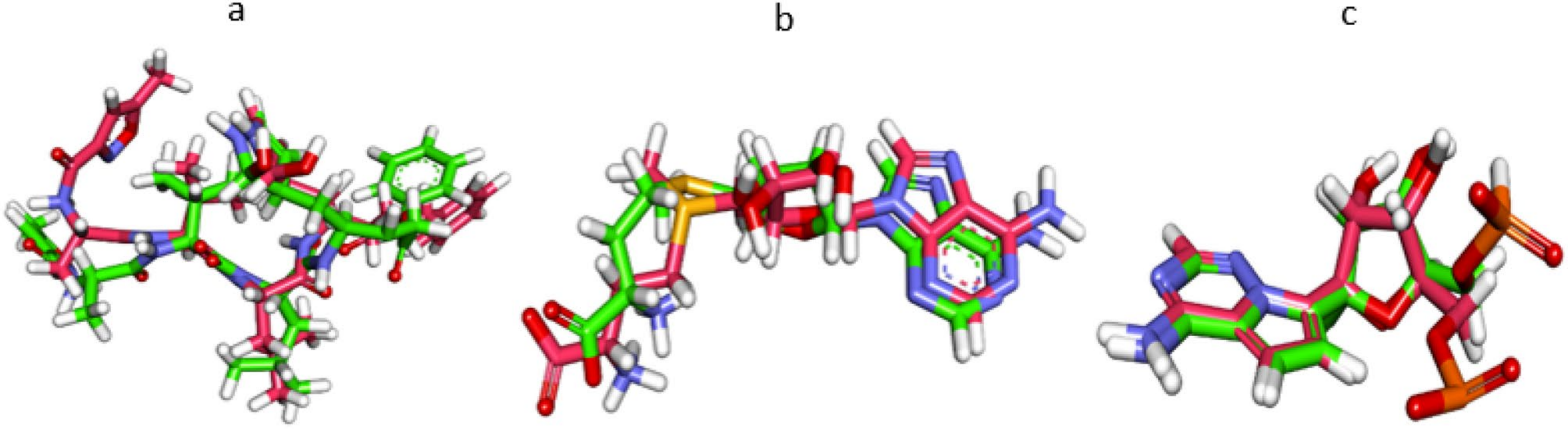
Superimposition of the co-crystallized pose and the docking pose of the same ligands. (**a**) PRD_002214 of COVID-19 main protease, (**b**) SAM of NSP10, (**c**) F86 of RNA-dependent RNA polymerase.

Re-docking processes of the co-crystallized ligands (PRD_002214, SAM, and F86) were preceded against the active pockets of COVID-19 main protease, NSP10, and RNA-dependent RNA polymerase, respectively, to validate the docking procedure. The calculated RMSD values between the re-docked poses and the co-crystallized ones were 3.10, 1.07, and 1.34, indicating the efficiency and validity of the docking processes (Fig. 2).

### Determination of anti-SARS-CoV2, MTT cytotoxicity, and anti-inflammatory activity

The percent inhibition of various concentrations of the isolated compound against SARS-CoV2 is shown in (Table 1). Interestingly, the isolated compound showed 95% viral inhibitory effects on SARS-CoV2 at a concentration of 5 ng/µl (12.48 µM).

**Table 1:** Anti-SARS-CoV2 activity of the isolated compound (Plaque Reduction Assay).

Conc (ng/µl)	Initial viral count (PFU/ml)	Viral count (PFU/ml)	Inhibition %
5	11 × 10^5^	0.55 × 10^5^	95
2.5		0.88 × 10^5^	92
1.25		1.32 × 10^5^	88
0.625		1.65 × 10^5^	85

**Figure 3:**
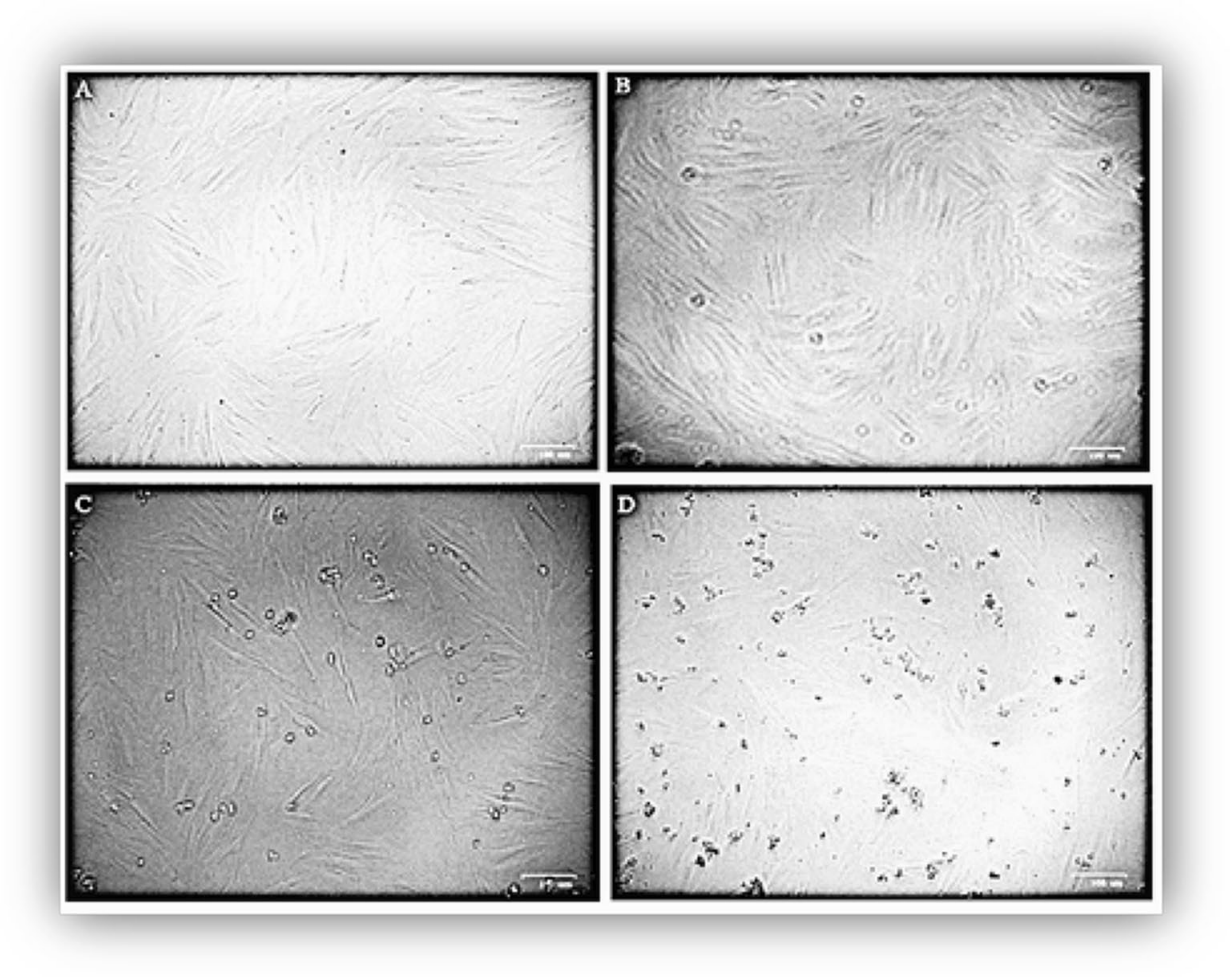
Morphology of fibroblast cells; (**a**) Control cells, (**b**) Cells treated with 3.12 μg/ml, (**c**) 6.25 μg/ml, (**d**) 12.5 μg/ml of the isolated compound.

In this study, human gingival fibroblast cell lines (Fig. 3) were exposed to different concentrations of the isolated compound. The cellular responses were determined using in vitro toxicity MTT assay. The changes in the morphology of cells exposed to different concentrations of the isolated compound were monitored using inverted microscopy. The results demonstrated that the cells exposed to 12.5 μg/ml of the isolated compound gradually lost their characteristic phenotype, started to shrink, and obtained irregular shapes. Percentage inhibition of the normal fibroblast cell growth at different concentrations of the isolated compound is shown in (supplementary Fig. S9), with an IC_50_ value of 11.35 ± 1.5 μg/ml (0.02 mM).

The anti-inflammatory activity of the isolated compound of different concentrations (10, 40, 100, 400, and 600 μg/ml) is shown in (Table 2).

**Table 2:** The anti-inflammatory activity of the isolated compound.

Concentration (μg/ml)	HRBC membrane stabilization	
	Isolated compound	Diclofenac
10	99.01	95.18
25	98.95	95.06
50	97.81	94.84
75	96.59	94.08
100	96.31	93.21
IC_50_ (μg/ml)	51.92 ± 0.03^b^	53.64 ± 0.01

Reported values are the mean ± SD (n = 3). Means in the same raw followed by different lower case letters are significantly different (p < 0.05).

## Discussion

Chemical studies on *Ophiocoma dentata* have resulted in discovering a new steroid, 5α-cholesta-4(27), 24-dien-3β, 23 β-diol. The structure of the isolated compound was determined with the help of spectroscopic studies. The isolated compound's NMR spectra matched those of 5 α-cholesta-9(11),24- dien-3 β,6 α,20β-triol-23-one 3-sulphate, reported by Yang et al.^16^, except for an absence of hydroxyl group at C-6, C-20 position and the double bond between C-9 and C-11 as well as additional double bond at C-4 and this was confirmed by the HMBC experiment. Inspection of its NMR data indicated a considerable similarity to those of ophidianoside F^17^ except for an absent hydroxyl group at C-6, C-20 and the double bond between C-9 and C-11, and the presence of a double bond between C-24 and C-25 position.

Docking studies were carried out using MOE14.0 software, yielding free energy (ΔG) values that indicate the examined molecule's binding interaction with the selected protein.

The isolated compound demonstrated good binding affinities with COVID-19 main protease (ΔG = − 24.68 kcal/mol), nsp10 (ΔG = − 23.47 kcal/mol), and RNA-dependent RNA polymerase (ΔG = − 29.86 kcal/mol), compared to the co-crystallized ligands PRD_002214 (ΔG = − 27.72 kcal/mol), SAM (ΔG = − 17.86 kcal/mol), and F86 (ΔG = − 23.56 kcal/mol), respectively (Table 3).

**Table 3:** The docking binding free energies of the isolated compound and the co-crystallized ligands against SARS-CoV-2 target proteins.

	COVID-19 main protease	NSP10	RNA-dependent RNA polymerase
The isolated compound	− 24.68	− 23.47	− 29.86
Co-crystallized ligand (PRD_002214)	− 27.72	–	–
Co-crystallized ligand (SAM)	–	− 17.86	–
Co-crystallized ligand (F86)	–	–	− 23.56

The docking mode of the co-crystallized ligand (PRD_002214) and COVID-19 main protease formed four hydrogen bonds and three hydrophobic interactions. The first pocket of M^pro^ was occupied by 2-oxopyrrolidin-3-yl moiety, forming two hydrogen bonds with Thr190 and Gln189. Additionally, the isopropyl moiety occupied the second pocket of M^pro^ forming three hydrophobic interactions with His41 and Met165. Furthermore, the third pocket was occupied by the benzyl acetate moiety, whereas the 5-methylisoxazole-3-carboxamide moiety was buried in the fourth pocket, forming a hydrogen bond with Thr26. Finally, one hydrogen bond was formed between an amide group and Met165 (supplementary Fig. S10).

Moreover, the co-crystallized ligand (SAM) formed three hydrogen bonds and seven hydrophobic interactions against the COVID-19 nsp10 protein. In detail, the tetrahydrofuran-3,4-diol moiety formed three hydrogen bonds with Asn6899, Tyr6930, and Asp6928. Besides, the 9H-purin-6-amine moiety formed three hydrophobic interactions with Phe6947 and Leu6898. Also, the (S)-(3-amino-3-carboxypropyl) dimethylsulfonium moiety formed many hydrophobic, electrostatic, and hydrogen bonding interactions with Asp6897, Lys6968, Lys6844, and Asp6928 (supplementary Fig. S11).

With respect to the binding mode of the co-crystallized ligand (F86) against COVID-19 RNA-dependent RNA polymerase, it formed three hydrogen bonds, six hydrophobic interactions, and two electrostatic interactions. Furthermore, the pyrrolo[2,1-f] [1,2,4] triazin-4-amine moiety formed six hydrophobic interactions with Urd20, Ade11, Arg555, and Val557. As well, the sugar moiety formed a hydrogen bond with Asp623. Additionally, the phosphate derivative moiety formed two electrostatic interactions and a hydrogen bond with Asp760, Asp623, and Cys622 (supplementary Fig. S12).

**Figure 4:**
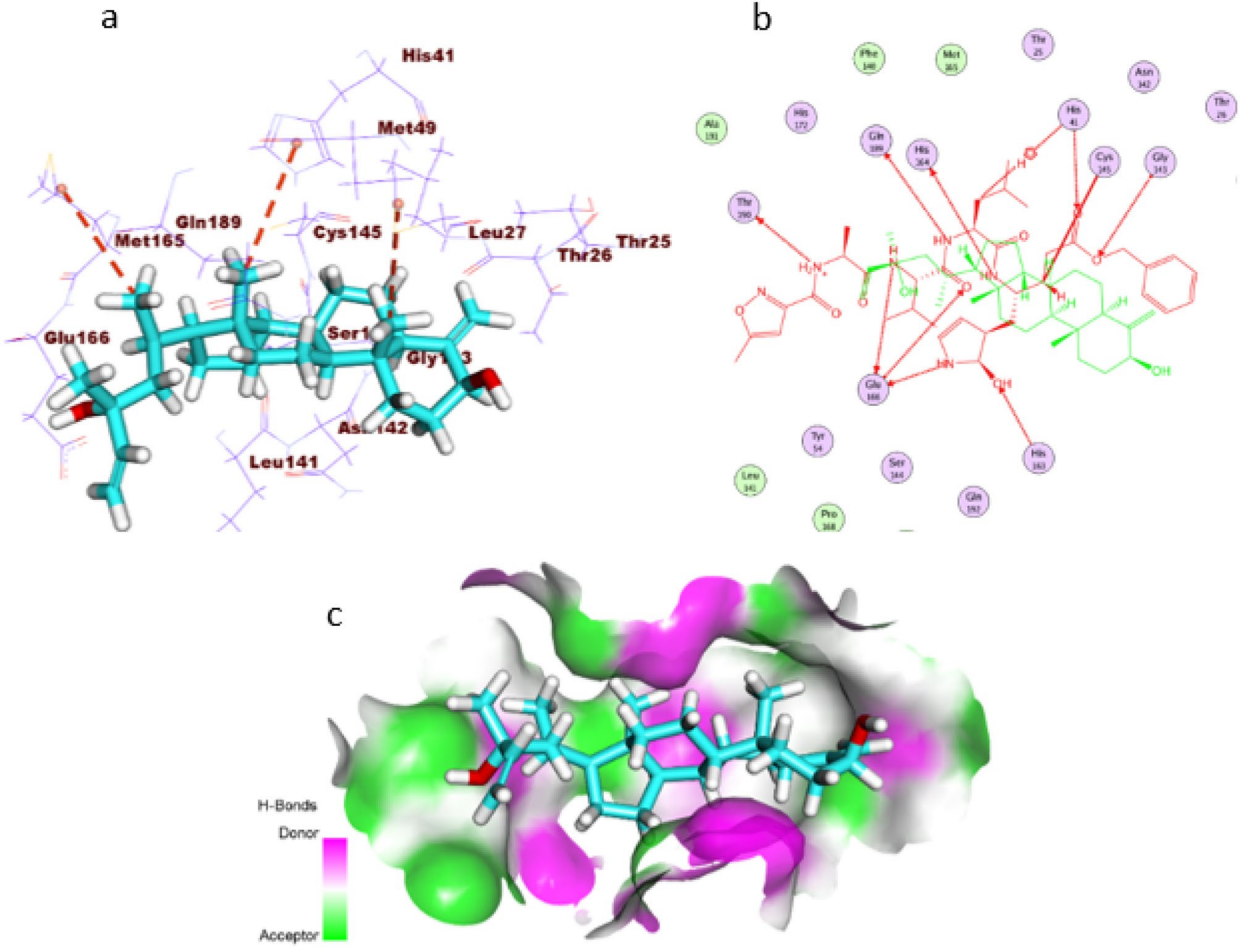
(**a**) 3D of the isolated compound docked into the active site of COVID-19 main protease. (**b**) 2D of the compound docked into the active site of COVID-19 main protease and superimposed with the co-crystallized ligand. (**c**) Mapping surface showing the compound occupying the active pocket of COVID-19 main protease.

Considering the isolated compound's binding mode against the COVID-19 main protease protein, it occupied three pockets of the protein with a similar orientation to that of the co-crystallized ligand inside the active pocket of M^pro^. Exhaustively, the (S)-2-methylenecyclohexan-1-ol moiety occupied the first pocket of M^pro^, forming one hydrophobic interaction with Met49. Moreover, the (3aR,7aS)-3a-methyloctahydro-1H-indene moiety was buried in the second pocket, forming a hydrogen bond with His41. In addition, the (R)-3-methylhex-1-en-3-ol moiety was incorporated in hydrophobic interaction with Met165 in the third pocket of M^pro^ (Fig. 4).

**Figure 5:**
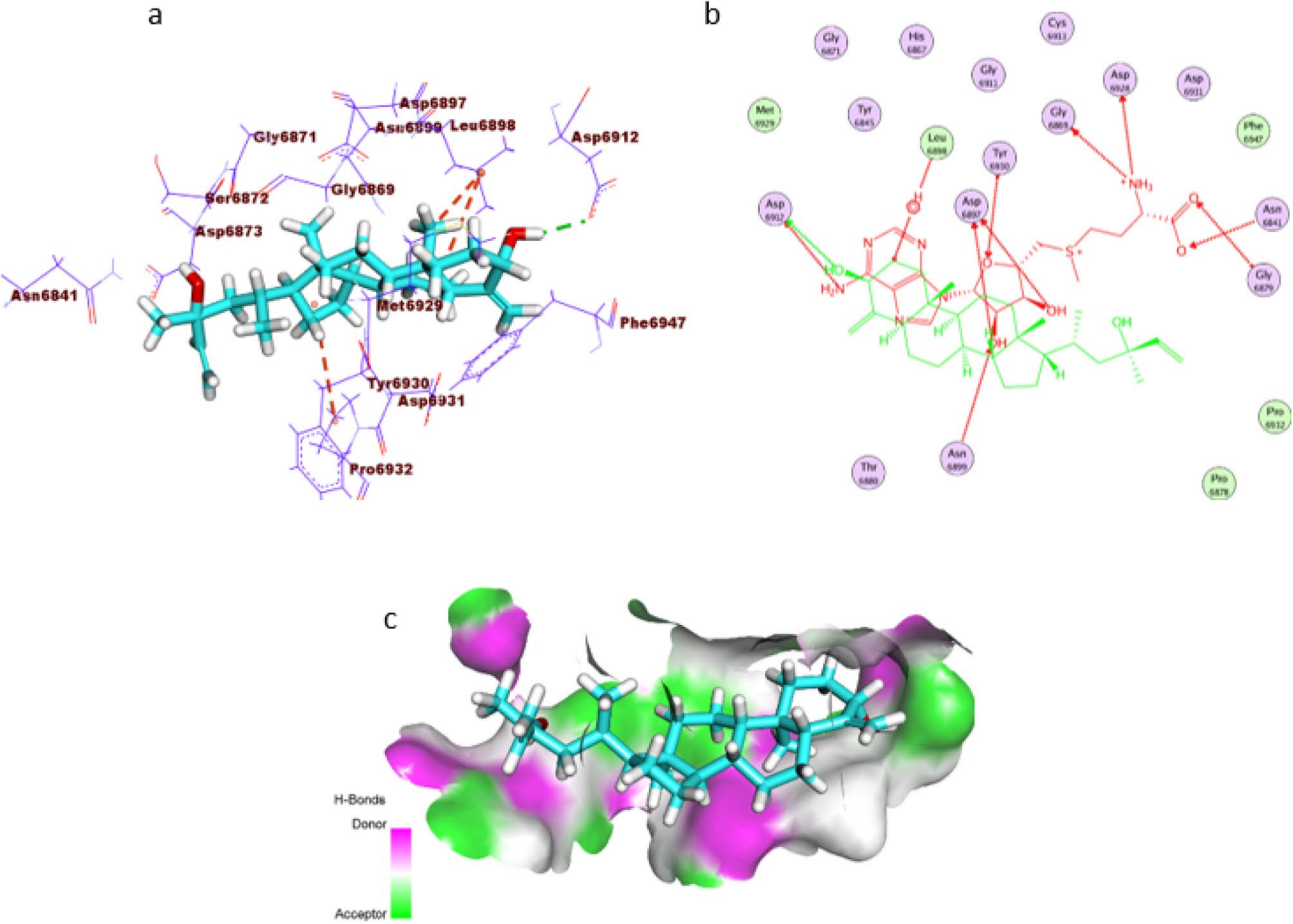
(**a**) 3D of the compound docked into the active site of COVID-19 NSP10. (**b**) 2D of the compound docked into the active site of COVID-19 NSP10 and superimposed with the co-crystallized ligand. (**c**) Mapping surface showing the compound occupying the active pocket of COVID-19 NSP10.

The compound’s interaction with the active pocket of NSP10 formed three hydrophobic interactions and a hydrogen bond. Closely, the hydroxyl group of (S)-2-methylenecyclohexan-1-ol moiety formed a hydrogen bond Asp6912. Such moiety formed two hydrophobic interactions with Leu6898. In addition, the (3aR,7aS)-3a-methyloctahydro-1H-indene moiety formed one hydrophobic interaction with Pro6932. The orientation of the compound inside the active pocket is similar to that of the co-crystallized ligand to some extent (Fig. 5).

**Figure 6:**
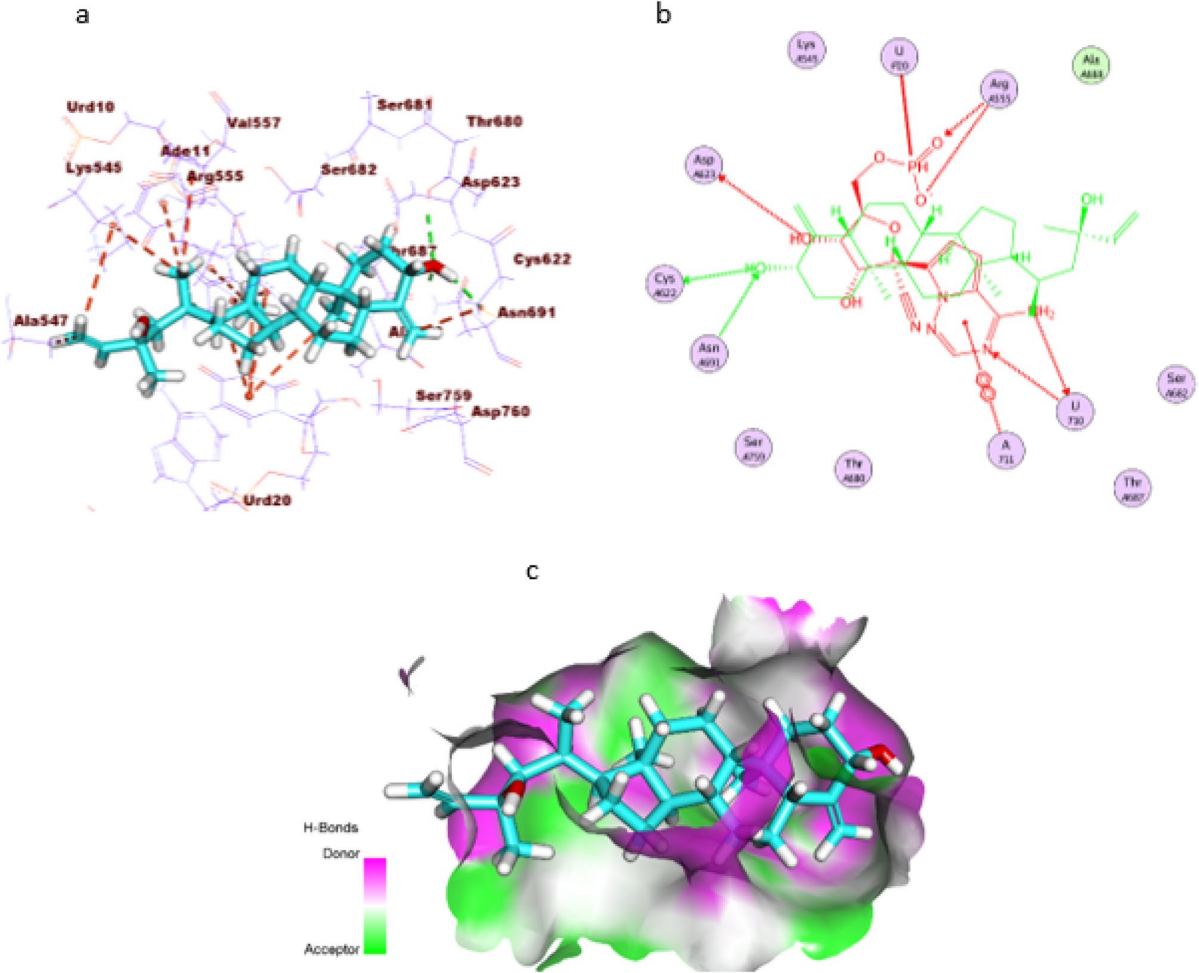
(**a**) 3D of the isolated compound docked into the active site of COVID-19 RNA-dependent RNA polymerase. (**b**) 2D of the compound docked into the active site of COVID-19 RNA-dependent RNA polymerase and superimposed with the co-crystallized ligand. (**c**) Mapping surface demonstrating the compound occupying the active pocket of COVID-19 RNA-dependent RNA polymerase.

The binding mode of the compound against RNA-dependent RNA polymerase formed twelve hydrophobic interactions and two hydrogen bonds with the active pocket. Comprehensively, the hydroxyl group of (S)-2-methylenecyclohexan-1-ol moiety formed two hydrogen bonds with Thr680 and Cys622. Such moiety formed a hydrophobic interaction with Urd20. Additionally, the (3aR,7aS)-3a-methyloctahydro-1H-indene moiety formed five hydrophobic interactions with Urd20 and Ade11, whereas the (R)-3-methylhex-1-en-3-ol moiety formed five hydrophobic interactions with Lys545, Urd10, Val557, and Ala547 (Fig. 6).

ADMET studies were carried out for the isolated compound using lopinavir as a reference compound. Discovery studio 4.0 was used to predict the following ADMET descriptors; blood–brain barrier (BBB) penetration, aqueous solubility, intestinal absorption, hepatotoxicity, cytochrome P450 inhibition, and plasma protein binding. The predicted descriptors are listed in (Table 4).

**Table 4:** The predicted ADMET for the isolated compound and lopinavir.

	BBB level^a^	Solubility level^b^	Absorption level^c^	CYP2D6 prediction^d^	Hepatotoxicity	PPB prediction^e^
The isolated compound	0	1	0	False	False	True
Lopinavir	4	3	2	False	True	True

^a^0 = very high, 1 = high, 2 = medium, 3 = low, 4 = very low.

^b^1 = very low, 2 = low, 3 = good, 4 = optimal.

^c^0 = good, 1 = moderate, 2 = poor, 3 = very poor.

^d^TRUE = inhibitor, FALSE = non inhibitor.

^e^FALSE means less than 90%, TRUE means more than 90%.

**Figure 7:**
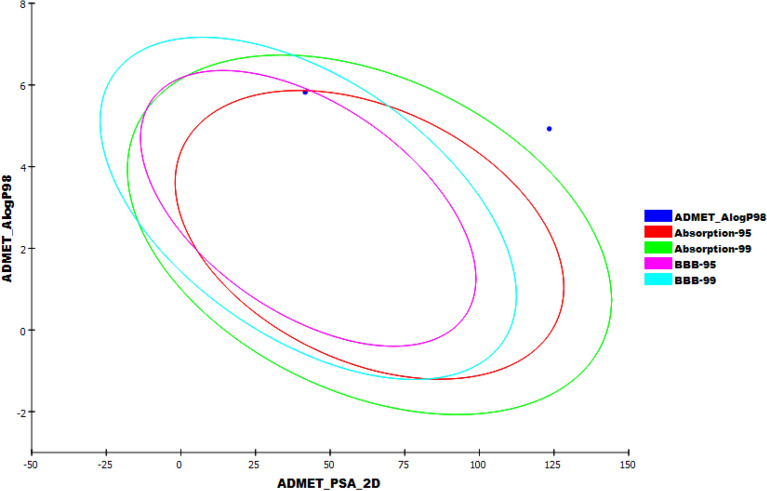
The expected ADMET study.

ADMET -BBB penetration studies predicted that the isolated compound possesses a high level compared to lopinavir, which is very low level. Although the compound showed a very low level of ADMET aqueous solubility, it was predicted to have a good level of intestinal absorption. The isolated compound was predicted to be a non-hepatotoxic and non-inhibitor of CYP2D6. Consequently, the liver dysfunction side effect is not expected upon administration of this compound. The plasma protein binding model predicts that the isolated compound can bind plasma protein over 90% (Fig. 7).

The isolated compound's toxicity was predicted using the validated and constructed models in the Discovery studio 4.0 software^18,19^ as follows: (i) FDA rodent carcinogenicity which expects the probability of a molecule to be a carcinogen. (ii) The tumorigenic dose rate 50 (TD50) of a chemical in a rodent chronic exposure toxicity test is predicted by the carcinogenic potency TD50^20^. (iii) The rat maximum tolerated dose (MTD) of a chemical is estimated using Rat maximum tolerated dose^21,22^. (iv) In the toxicity test of a chemical, the rat oral LD50 predicts the rat oral acute median lethal dose (LD50)^23^. (v) The rat chronic LOAEL predicts a compound's rat chronic lowest observed adverse effect level (LOAEL) value^24,25^. (vi) Ocular irritancy predicts if a certain compound is probable to be an ocular irritant and how severe the irritation will be in the Draize test^26^. (vii) skin irritancy predicts if a compound would irritate the skin and how severe it will be in a rabbit skin irritancy test^26^.

The isolated compound exhibited in silico low adverse effects and toxicity towards the tested models as shown in (Table 5). About FDA rodent carcinogenicity, the tested compound was predicted to be non-carcinogenic. For carcinogenic potency TD_50_ rat model, the tested compound showed TD_50_ value of 0.621 mg/kg body weight/day which is less than lopinavir (TD_50_ = 3.553 mg/kg body weight/day). Regarding the rat maximum tolerated dose model, the tested compound showed a maximum tolerated dose value of 0.040 g/kg body weight which is less than lopinavir (0.117 g/kg body weight). The examined compound demonstrated an oral LD_50_ value of 1.715 mg/kg body weight/day which is higher than lopinavir (1.154 mg/kg body weight/day). For the rat chronic LOAEL model, the isolated compound showed a LOAEL value of 0.002 g/kg body weight which is less than lopinavir (0.049 g/kg body weight). Moreover, the compound was predicted to be an irritant in the ocular irritancy model. Finally, it was expected to have moderate irritancy against the skin irritancy model.

**Table 5:** The isolated compound's toxicity properties.

	FDA rodent carcinogenicity (rat-male)	Carcinogenic potency TD_50_ (rat)^a^	Rat maximum tolerated dose (feed)^b^	Rat oral LD_50_^b^	Rat chronic LOAEL^b^	Ocular irritancy	Skin irritancy
The isolated compound	Non-carcinogen	0.621	0.040	1.715	0.002	Irritant	Moderate
Lopinavir	Non-carcinogen	3.553	0.117	1.154	0.049	Irritant	None

^a^Unit: mg/kg body weight/day.

^b^Unit: g/kg body weight.

According to Mostafa et al.^27^, Azithromycin, which is an FDA-approved antimicrobial drug with promising antiviral activity for repurposing against COVID-19, its mode of action was during the viral replication, which demonstrated up to 70% inhibition at concentration 10.4 µM and exhibited moderate virucidal effect with 37% viral inhibitory effect. Additionally, the Niclosamide drug exhibited a high virucidal effect with a 78% viral inhibitory effect at concentration 10.4 µM, suggesting that our isolated compound is a promising viral inhibitor against SARS-CoV2 and its mode of action could be during the viral replication or had a virucidal effect.

The results revealed that the isolated compound has low toxicity to normal cells and could be used as an antiviral agent after performing in vivo assays. Interestingly, the effective dose which inhibited 95% of the viral count was 5 ng/µl (5 μg/ml) and that below the IC_50_ value (11.35 ± 1.5 μg/ml) of the normal fibroblast cells. The obtained IC_50_ for the isolated compound was lowered than the reference drug (Diclofenac), indicating that the compound was more potent than diclofenac and could be used as an anti-inflammatory drug.

Echinoderms are rich bioactive components that provide tremendous pharmaceutical and clinical medicine values. Similarly, like many marine invertebrates, survival requirements have led to the evolution of these complex substances. Interestingly, several secondary metabolites were proven to have a potent antiviral activity as well as a relatively non-cytotoxicity. As a result, these promising echinoderm natural compounds could be used to develop new antiviral drugs. In this study, 5α-cholesta-4(27), 24-dien-3β, 23 β-diol was isolated from the brittle stars, *Ophiocoma dentata* showing 95% inhibition of COVID-19 virus at concentration 5 ng/µl, which is safe to the normal cells. The current study's findings are promising regarding finding an effective cure for the COVID-19 pandemic. Further in-vivo studies regarding the clinical and structure–activity relationship are needed to confirm the isolated compound's potential against the infectious virus SARS-CoV-2.

Marine natural products remain a promising source for discovering high structural diversity and various bioactivities that can be directly developed or used as starting points for the development of novel medications. In the future, we must consider the final dosage form for human use (e.g., tablets, capsules, and injections) to have the potential to be translated into a clinically applicable therapeutic. Biological challenges, large-scale manufacturing, biocompatibility, intellectual property, stable storage, government regulations, and overall cost-effectiveness should all be considered during clinical development. Future research could focus on determining the biodistribution, Pharmacodynamics, and pharmacokinetics in humans, as well as screening for safety and demonstrating the preliminary efficacy of the isolated compound. It is critical to comprehend how the in-vivo environment influences its long-term structural organization, retention, and clearance.

## Methods

### Sample collection

Live Brittle star; *O. dentata* with body weight (50–200 g) were collected in June 2019 from the Red Sea shore at Hurghada, Egypt, from the tidal zone in front of the Marine biological station (NIOF), at about 4–10 m deep between latitude 27.28°N and longitude 33.77° E. This species is among the most abundant brittle star observed in the area. Samples were identified according to their morphological characteristics with taxonomic reference^28^. Voucher specimens (2019-618MS) have been deposited at the National Institute of Oceanography and Fisheries, Egypt. Samples were washed, packed in polypropylene bags, and immediately frozen before being transported to the laboratory and stored at − 20 °C until use.

### Extraction and isolation

The whole bodies of brittle star *O. dentata* (1 kg) were extracted with CHCl_3_/MeOH at room temperature three times then concentrated under reduced pressure until they were dried. The resultant residue was kept frozen at − 20 °C for further purification and analysis. The crude extract (14.62 g) of brittle star (*O. dentata*) was subjected to (1^ry^) column packed with silica gel size of 60–200 µm (70–230 mesh) and elution via mobile phase Hexane: DCM (100:0%) then (95:5%) and increasing the polarity until reaching (0:100%). Consequently, 250 fractions were collected, and fractions with the same RF value were combined to give 32 fractions, as shown in supplementary Fig. S1.

Finally, we isolated a new compound namely 5α-cholesta-4(27), 24-dien-3β, 23 β-diol (Fig. 1) from *O. dentata* with TLC (DCM: MeOH, 99:1 v/v): Rf = 0.53; 1H NMR (400 MHz, CDCl_3_): δ 1.05 (m, 2H), 1.80 (m, 2H), 1.06 (d, J = 10 Hz, 3H), 2.04 (d, J = 8.8 Hz, 2H), 5.93 (m, 1H), 5.09 (d, J = 10.8 Hz, 2H), 5.23 (d, J = 17.2 Hz, 2H), 1.44 (s, 3H), 4.67 (s, 2H), 4.93 (s,2H); 13C NMR (125 MHz, CDCl_3_): δ 145.6, 145.1, 111.7, 109.1, 73.3, 67.3, 53.2, 46.1, 41.3, 37.1; IR: 3424 cm^−1^, 1742 cm^−1^; HREIMS (m/z): C_27_H_44_O_2_, 400.6470.

### Spectroscopic characterization of the isolated compound

#### NMR analysis

The ^1^H NMR and ^13^C NMR analysis was measured at Bruker AVANCE 400 MHZ NMR spectrometers at the University of Winnipeg, Canada, using Deuterated chloroform (CDCl_3_). COSY, HSQC, HMBC, and NOESY were also measured. Chemical shift (δ) values were given in *ppm* while coupling constants (J) in *Hz*.

#### FT-IR analysis

The compound's functional groups were identified using infrared spectroscopy. The powdered compound was mixed 1:1 with dried KBr powder to make a compressed pellet using the Bruker Alpha Fourier transform infrared spectroscopy spectrometer with the subsequent recording of infrared spectrum in the transmission mode from 4000 to 400 cm^−1^.

#### GC–MS analysis

The compound was subjected to GC–MS to identify and confirm the structure of the purified bioactive compound. The sample was analyzed in an Agilent 7890A-5975C high-resolution gas chromatography-mass spectroscopy instrument. The carrier gas was Helium, and the analysis was performed at 90–300 °C. The identification and confirmation of the structure of the compound were made using computer matching of spectral data with that of standards.

### Molecular docking analysis of isolated compound

The target proteins' crystal structures: (i) Main protease of COVID-19 (M^pro^) (PDB ID: *6*lu7, resolution: 2.16 Å), (ii) non-structural protein (nsp10) (PDB ID: 6W4H, resolution: 1.80 Å), and (iii) RNA-dependent RNA polymerase (PDB ID: 7BV2, resolution: 2.50 Å) were downloaded from Protein Data Bank (http://www.pdb.org). Molecular Operating Environment (MOE) was used for the docking analysis^29^. The free energies and binding modes of the tested molecules against target proteins were established in this investigation. Water molecules were first eliminated from target proteins crystal structures, leaving only one chain required for binding. The reference ligands were co-crystallized. The hydrogen atoms were then hidden, and the protein structures were protonated. Afterward, the MMFF94x force field was used to reduce the energy. After that, the binding pockets of each protein were defined^30–32^.

ChemBioDraw Ultra 14.0 was used to draw the structure of the investigated compound and the co-crystallized ligands, which were saved as SDF formats. Subsequently, the stored files were opened using MOE software, and 3D structures were protonated. The MMFF94x force field was then applied to minimize the energy of the molecules. Validation processes for each target receptor were carried out by running the docking process for only the co-crystallized ligand. Valid performance is indicated by low RMSD values between docked and crystal conformations^33,34^. The output from the MOE software was further analyzed and visualized using the Discovery Studio 4.0 software^35–38^.

### In silico ADMET analysis

Discovery studio 4.0 was used to determine the compound's ADMET descriptors (absorption, distribution, metabolism, excretion, and toxicity). According to the small molecule preparation protocol, the compound was prepared and minimized after applying the CHARMM force field. These studies were then carried out using the ADMET descriptors procedure^33,36^.

### In silico toxicity studies

Discovery studio 4.0 was used to calculate the toxicity parameters of the isolated compound. Lopinavir was used as a reference drug. The toxicity prediction (extensible) protocol calculated various parameters^36,39,40^.

### Determination of anti-SARS-CoV2 activity

In order to evaluate the antiviral activity of the isolated compound, the plaque reduction assay was carried out according to Mustafa et al.^27^ at the main laboratories of chemical warfare in Egypt. Percentage reduction in plaques formation in comparison to control wells was recorded as follows:

$$ \% \;{\text{inhibition}} = \frac{{{\text{viral count}}\;\left( {{\text{untreated}}} \right) - {\text{viral count}}\;\left( {{\text{treated}}} \right)}}{{{\text{viral count}}\;\left( {{\text{untreated}}} \right)}} \times 100 $$

### MTT cytotoxicity assay

MTT assay, an in-vitro cytotoxicity test, was used to measure cell viability and membrane damage following van de Loosdrecht et al.^41^. Sample dissolved in 10% DMSO, then tested against human gingival fibroblast cell lines (obtained as a gift from faculty of medicine, Egypt) by using 3-(4,5-dimethylthiazol-2-yl)-2,5-diphenyltetrazolium bromide (MTT). A microplate reader (ASYS-EXPERT 96) was utilized to measure the optical density at 540 nm. The changes in the morphology of cells exposed to the isolated compound were monitored using inverted microscopy and Biorad (ZOE fluorescent cell imager).

### Anti-inflammatory activity

The isolated compound's anti-inflammatory activity in-vitro was assessed by the human red blood corpuscles (HRBCs) membrane-stabilizing method^42^. The percentage membrane stabilization was calculated using the following formula:

$$ \% \;Protection = 100 - \frac{OD\;of\;test}{{OD\;of\;control}} \times 100 $$

### Statistical analysis

SPSS version 16 was used to analyze the data obtained using one-way analysis of variance (ANOVA). Values are expressed as mean ± SD (n = 3). P values ≤ 0.05 were considered significant.

## Supplementary Information

Supplementary Information.

## Data Availability

All data generated or analyzed during this study are included in this published article (and its Supplementary Information files).
