# Supplementary material for: Characterization, in-silico, and in-vitro study of a new steroid derivative from Ophiocoma dentata as a potential treatment for COVID-19
Source: Sci Rep. 2022 Apr 7;12:5846. doi: 10.1038/s41598-022-09809-2 (PMC8991244; doi:10.1038/s41598-022-09809-2)
Supplement: Supplementary file 1 — Supplementary Information. [file 41598_2022_9809_MOESM1_ESM.docx]

**Supplementary material**

**Characterization, *in-silico*, and *in-vitro* study of a new steroid derivative from *Ophiocoma dentata* as a potential treatment for COVID-19**

Mohamed S.M. Abd El Hafez***^1, 2^**, Miral G. AbdEl-Wahab**^2^**, Mohamed G.Seadawy**^3^**, Mostafa F. El-Hosseny**^3^**, Osama Beskales**^4^**, Ali saber ali abdel-hamid**^2^**, Maha A. El Demellawy**^2,5^**, Doaa A. Ghareeb**^2,6,7^**

**^1^** National Institute of Oceanography and Fisheries (NIOF), Egypt.

**^2^** Center of Excellence for Drug Preclinical Studies (CE-DPS), Pharmaceutical and Fermentation

Industries Development Centre (PFIDC), City of Scientific Research and Technological Applications

(SRTA-City), Egypt.

**^3^** Biological Prevention Department, Egyptian Army, Egypt.

**^4^** Medical services department, The Egyptian army, Egypt.

**^5^**Medical Biotechnology Department, Genetic Engineering & Biotechnology Research Institute, City of

Scientific Research and Technological Applications (SRTA-City), Egypt.

**^6^** Bio-screening and Preclinical Trial Lab, Biochemistry Department, Faculty of Science, Alexandria

University, Alexandria, Egypt.

**^7^** Biochemistry Department, Faculty of Science, Alexandria University, Alexandria, Egypt.

^*^Corresponding Author: [mohamedsaid80@yahoo.com](mailto:mohamedsaid80@yahoo.com)

**ABSTRACT**

The medicinal potential of marine invertebrates' bioactive components that may act as anti-COVID-19 demonstrated promising results. *Ophiocoma dentata*, which is common in the Red Sea, is one such source. Therefore, this study aimed to isolate a new compound from the brittle star, *Ophiocoma dentata*, and evaluate its efficacy as anti-COVID-19 *in-silico* and *in-vitro*. Standard procedures were followed in order to assess the isolated compound’s preliminary toxicity and anti-inflammatory properties. Computer virtual screening technology through molecular docking and ADMET studies was conducted as well as a new steroid derivative was isolated for the first time, named 5α-cholesta-4(27), 24-dien-3β, 23 β-diol. Investigation of the Anti-Covid-19 activity of the isolated compound using a Plaque reduction assay revealed 95% inhibition at a concentration of 5ng/µl (12.48 µM). Moreover, this compound showed an IC_50_ of 11350±1500 ng/ml against the normal fibroblast cells, indicating its safety. Interestingly, this compound exhibited anti-inflammatory activity with an IC_50_ of 51.92±0.03 μg/ml compared to a reference drug’s IC_50_ of 53.64±0.01 μg/ml, indicating that this compound is a potent anti-inflammatory. *In silico* data have proved that the isolated compound is a promising viral inhibitor against SARS-CoV2 and is thus recommended as a future nature preventive and curative antiviral drug.

**Keywords**

SARS-CoV2; Molecular docking; Marine natural products; Brittle star; Red sea; NMR.

**Table S1.** ^1^H and ^13^C NMR data of the isolated compound.

**Figure S1.** Extraction flow chart of the isolated compound from the brittle star (*O. dentata*).

**Figure S2.** ^1^H NMR spectrum of the isolated compound.

**Figure S3.** ^13^C NMR spectrum of the isolated compound.

**Figure S4.** HSQC spectrum of the isolated compound.

**Figure S5.** HMBC spectrum of the isolated compound.

**Figure S6.** COSY spectrum of the isolated compound.

**Figure S7.** NOESY spectrum of the isolated compound.

**Figure S8.** FT-IR spectrum of the isolated compound.

**Figure S9.** Percentage inhibition of the normal fibroblast cell growth at different concentrations of the isolated compound.

**Figure S10**. (a) 3D of Co-crystallized ligand (PRD_002214) docked into the active site of COVID-19 main protease. (b) Mapping surface showing co-crystallized ligand (PRD_002214) occupying the active pocket of COVID-19 main protease.

**Figure S11**. (a) 3D of co-crystallized ligand **(**SAM**)** docked into the active site of COVID-19 NSP10. (b) Mapping surface showing co-crystallized ligand **(**SAM**)** occupying the active pocket of COVID-19 NSP10.

**Figure S12**. (a) 3D of co-crystallized ligand **(**F86**)** docked into the active site of COVID-19 RNA-dependent RNA polymerase. (b) Mapping surface showing co-crystallized ligand **(**F86**)** occupying the active pocket of COVID-19 RNA-dependent RNA polymerase.

**Table S1.** ^1^H and ^13^C NMR data of the isolated compound.

| Position | δ_H_ (*J* in HZ), mult. | δ_C_ |
| --- | --- | --- |
| 1 | 1.05(m) | 37.1 |
| 2 | 1.80(m) | 41.3 |
| 3 | 4.14(m) | 67.3 |
| 4 |  | 145.6 |
| 5 | 1.75(m) | 53.2 |
| 6 | 1.73(m) | 35.9 |
| 7 | 1.62(m) | 31.9 |
| 8 | 1.48(m) | 23.6 |
| 9 | 0.89(m) | 24.7 |
| 10 |  | 46.1 |
| 11 | 1.68(m) | 22.6 |
| 12 | 1.65(m) | 20.7 |
| 13 |  | 42.0 |
| 14 | 0.87(m) | 22.7 |
| 15 | 1.57(m) | 27.1 |
| 16 | 1.32(m) | 29.7 |
| 17 | 1.10(m) | 30.3 |
| 18 | 0.86(s) | 14.1 |
| 19 | 1.19(s) | 16.5 |
| 20 | 1.35(m) | 32.8 |
| 21 | 1.06(d,10.0) | 27.8 |
| 22 | 2.04(d,8.8) | 37.4 |
| 23 |  | 73.3 |
| 24 | 5.93(m) | 145.1 |
| 25 | 5.09(d,10.8)  5.23(d,17.2) | 109.1 |
| 26 | 1.44(s) | 28.4 |
| 27 | 4.93(s)  4.67(s) | 111.7 |

^1^H NMR at 400 MHz, ^13^C NMR at 100 MHz in CDCl_3_


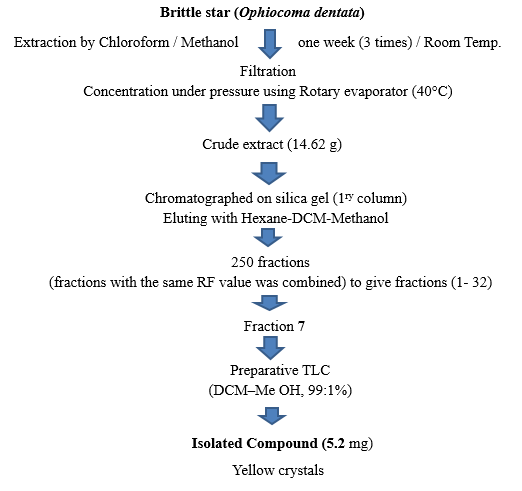


**Figure S1.** Extraction flow chart of the isolated compound from the brittle star (*O. dentata*).


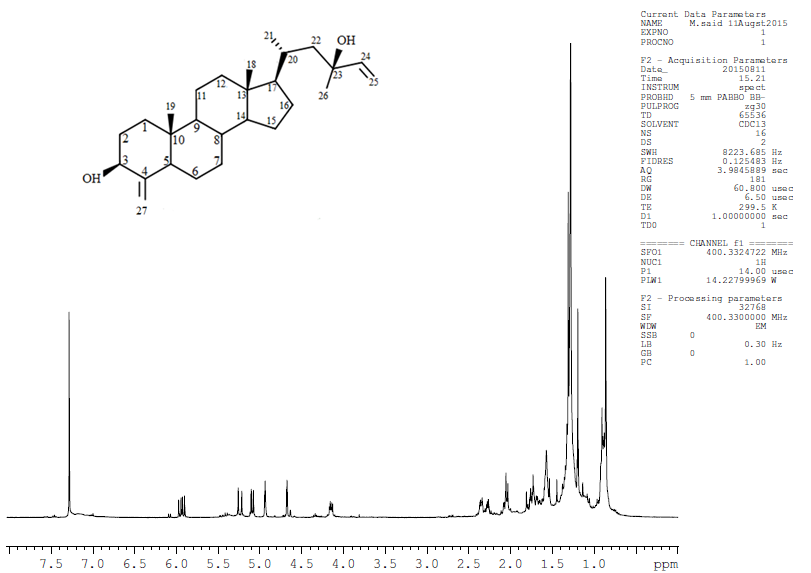


**Figure S2.** ^1^H NMR spectrum of the isolated compound.


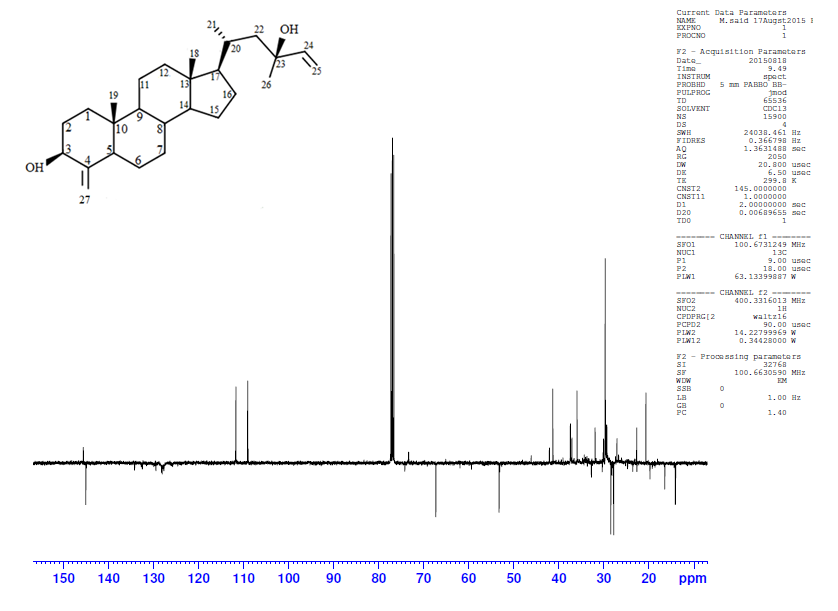


**Figure S3.** ^13^C NMR spectrum of the isolated compound.


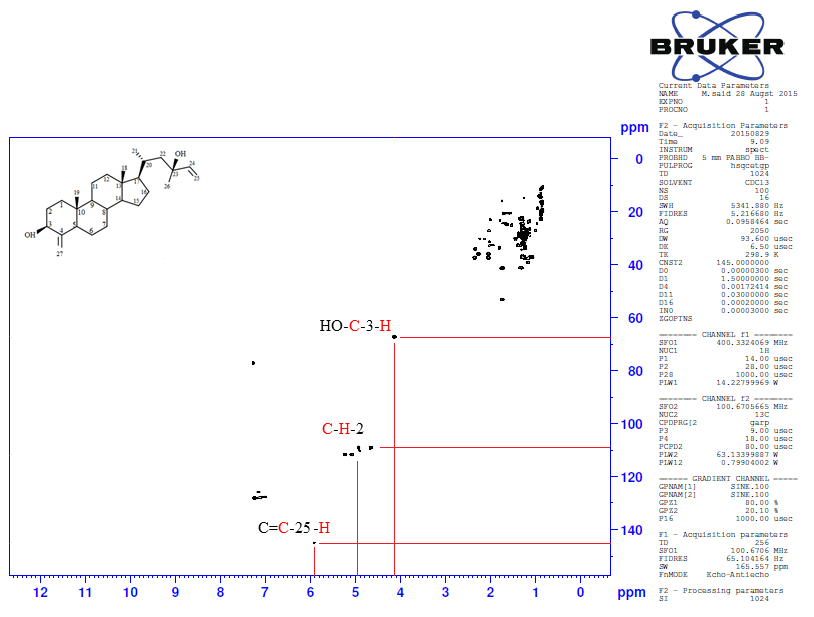


**Figure S4.** HSQC spectrum of the isolated compound.


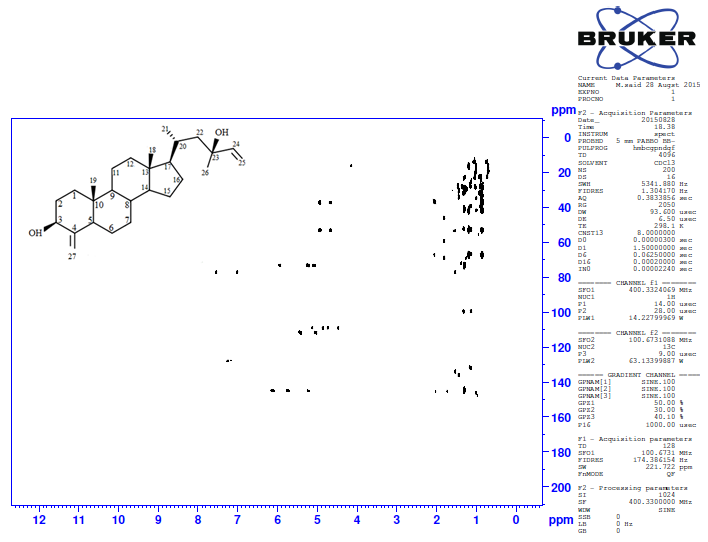


**Figure S5.** HMBC spectrum of the isolated compound.


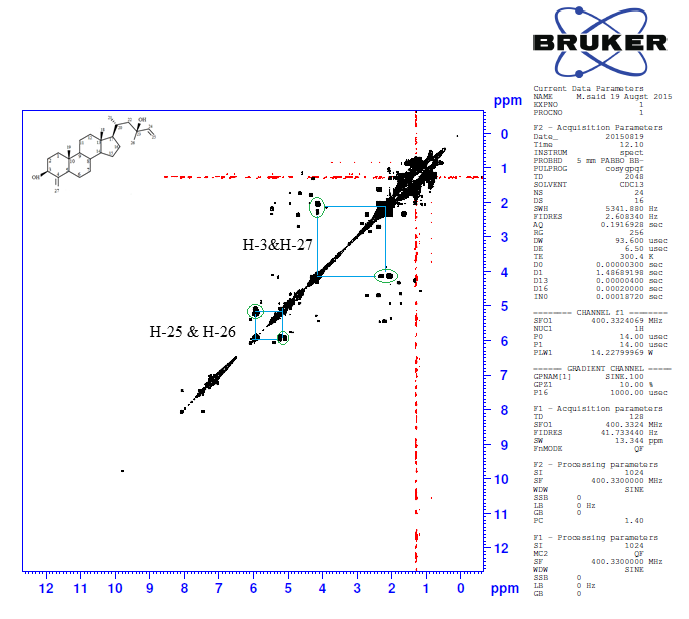


**Figure S6.** COSY spectrum of the isolated compound.

**Figure S7.** NOESY spectrum of the isolated compound.


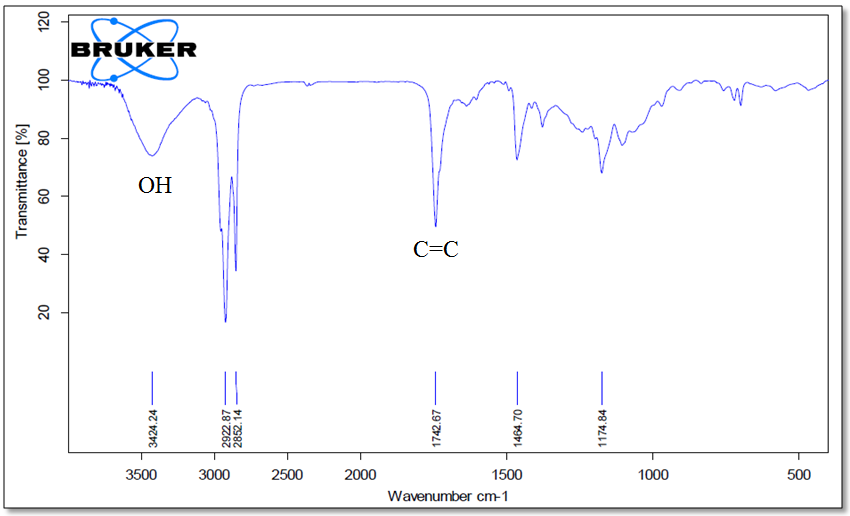


**Figure S8.** FT-IR spectrum of the isolated compound.


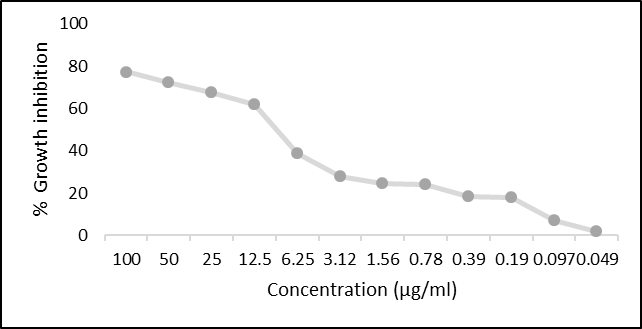


**Figure S9.** Percentage inhibition of the normal fibroblast cell growth at different concentrations of the isolated compound.


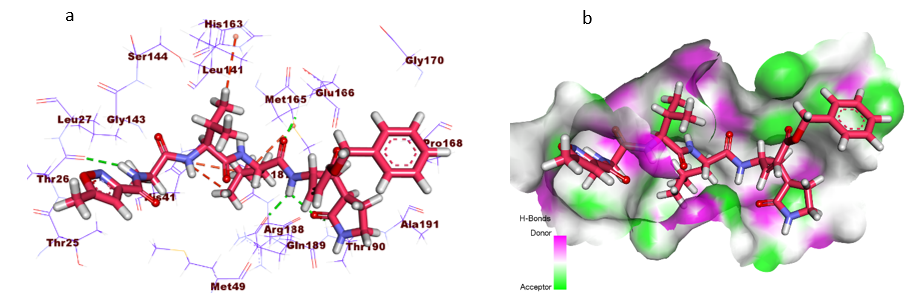


**Figure S10**. (a) 3D of Co-crystallized ligand (PRD_002214) docked into the active site of COVID-19 main protease. (b) Mapping surface showing co-crystallized ligand (PRD_002214) occupying the active pocket of COVID-19 main protease.


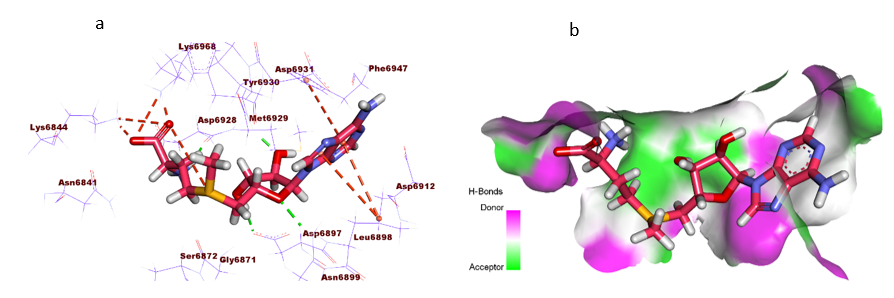


**Figure S11**. (a) 3D of co-crystallized ligand **(**SAM**)** docked into the active site of COVID-19 NSP10. (b) Mapping surface showing co-crystallized ligand **(**SAM**)** occupying the active pocket of COVID-19 NSP10.


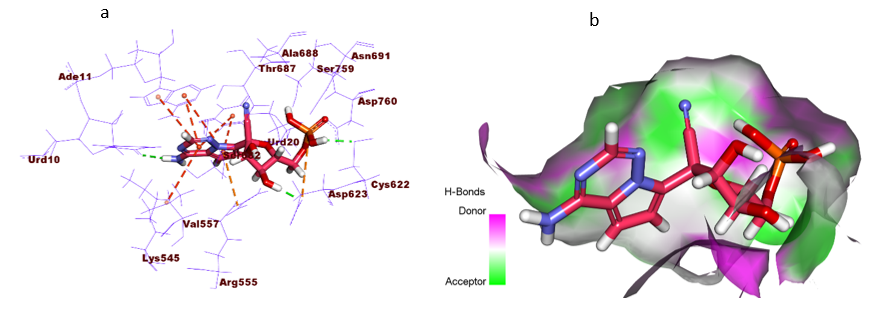


**Figure S12**. (a) 3D of co-crystallized ligand **(**F86**)** docked into the active site of COVID-19 RNA-dependent RNA polymerase. (b) Mapping surface showing co-crystallized ligand **(**F86**)** occupying the active pocket of COVID-19 RNA-dependent RNA polymerase.
